# Impact of Intra‐fractional respiratory motion on dose distribution in lattice radiotherapy for liver tumors

**DOI:** 10.1002/pro6.70044

**Published:** 2025-12-20

**Authors:** Kuo Li, Yong Yin, Tonghai Liu, Tianyuan Dai, Jian Zhu, Zhenjiang Li

**Affiliations:** ^1^ Department of Radiation Physics Shandong Cancer Hospital and Institute Shandong First Medical University and Shandong Academy of Medical Sciences Jinan China; ^2^ Department of Radiation Physics Technology Shandong Public Health Clinical Center Jinan China; ^3^ Shandong Provincial Key Medical and Health Laboratory of Pediatric Cancer Precision Radiotherapy (Shandong Cancer Hospital) Jinan China; ^4^ Centre de Recherche en Information BioMdicale Sino‐ français Nanjing China

**Keywords:** dose distribution, lattice radiotherapy, respiratory motion, valley‐to‐peak dose ratio

## Abstract

**Purpose:**

To quantify the detrimental impact of respiratory motion on the critical “peak‐and‐valley” dose distribution in lattice radiotherapy (LRT) for liver tumors, to thereby evaluate the necessity of motion management.

**Methods:**

This study assessed the data of 24 patients with liver cancer who underwent free‐breathing 4D‐CT simulation, for which a 2×2×2 vertices LRT tree was constructed. Volumetric Modulated Arc Therapy (VMAT) plans were generated based on the results of free‐breathing CT. The delivered dose distribution under respiratory motion was then simulated by dividing the planned dose into 10 equal subcomponents, applying phase‐specific isocenter shifts derived from 4D‐CT displacement measurements, and summing the deformed doses. The metrics analyzed included the vertex dose deviation, valley‐to‐peak dose ratio (VPDR), and low‐dose bath volumes.

**Results:**

The analysis revealed a mean 3D respiratory motion error of 8.59 mm, with predominant displacement in the superior‐inferior (SI) direction (6.78±3.15 mm). Respiratory motion significantly degraded the LRT dose distributions; specifically, the vertices mean dose (D_mean_) decreased from 50.19 Gy to 42.26 Gy, while the maximum dose (D_max_) from 58.94 Gy to 52.63 Gy. Crucially, the VPDR increased in all directions, with the most pronounced increase observed in the SI direction (0.14±0.04 to 0.18±0.06, representing a 28.6% increase), escalating exponentially when motion error exceeded 8 mm. Increases in the left‐right (LR) (0.41 to 0.44, increasing 7.3%) and antero‐posterior (AP) (0.41 to 0.45, increasing 9.8%) directions were comparatively smaller. The motion also paradoxically altered the low‐dose regions; while the absolute V_40%_, V_20%_, and V_10%_ volumes decreased by approximately 10 cm^3^ in the delivered plan, normalization to match the prescription coverage revealed significant increases in these volumes (minimum increases: V_40%_ by 3.59 cm^3^, V_20%_ by 15.85 cm^3^ and V_10%_ by 92.29 cm^3^).

**Conclusion:**

Respiratory motion severely degrades essential spatial fractionation during liver LRT, particularly when exceeding 8 mm and occurring predominantly in the SI direction. This motion reduces peak vertex doses, increases the VPDR (homogenizing the dose distribution), and disrupts the low‐dose bath volumes critical for normal tissue sparing and the bystander effect.

## Graphical abstract

This study quantitatively analyzed the impact of liver motion on lattice radiotherapy within a breathing cycle using 4D‐CT, providing a physical basis for precise patient treatment. Tumor motion does not cause target deviation in lattice radiotherapy, but it significantly adversely affects unique dose distribution metrics of spatially fractionated radiotherapy (SFRT), such as the valley‐to‐peak dose ratio (VPDR) and low‐dose areas. Motion management techniques improve the repeatability and sustainability of treatment, facilitating the effective implementation of lattice radiotherapy. However, this study theoretically explored tumor motion and still requires clinical validation to prove the sustainability of the results

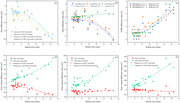

## 1 INTRODUCTION

Hepatocellular carcinoma (HCC) and metastatic liver tumors present unique challenges for radiotherapy due to the low radiation tolerance of the liver, with a tolerance dose 5/5 of 30 to 35 Gy versus the >60 Gy required for ablation.^1^, ^2^ Although stereotactic body radiotherapy (SBRT) achieves local control of small lesions (<5 cm), bulky tumors remain problematic due to the high risk of radiation‐induced liver disease (RILD).^3^

Spatially fractionated radiotherapy (SFRT), including the GRID and LATTICE techniques, delivers ultra‐heterogeneous “peak‐and‐valley” dose distributions. This design has two key biological advantages: (1) peak doses (D_peak_, >15 Gy/fraction) trigger vascular disruption and immunogenic cell death, releasing damage‐associated molecular patterns (DAMPs) that stimulate systemic immune responses^4^, ^5^; and (2) valley doses (D_valley_, <5 Gy/fraction) preserve stem cells and microvasculature in the normal parenchyma, enabling tissue regeneration.^6^ This approach can destroy the bulk of the tumor, while preserving the healthy tissue. By maintaining low‐dose regions, these patterns may stimulate the immune system to recognize and eliminate residual cancer cells. SFRT is typically administered with palliative intent, aiming to alleviate symptoms, such as mass effects and hemorrhage, while minimizing side effects. It is particularly suitable for debulking large hypoxic tumors that commonly exhibit inherent resistance to radiotherapy.

Studies have shown that the amplitude of the liver respiratory motion varies considerably among different patients, with an average motion amplitude of up to 9.25 mm.^7^ This motion can have a pronounced impact on radiotherapy dose distribution, particularly for intensity‐modulated radiotherapy (IMRT) and proton therapy.^8^ Furthermore, liver motion may increase the radiation dose to the normal tissues, thereby increasing the risk of RILD.^9^, ^10^ Although extensive research over the past few years has focused on respiratory‐induced tumor motion during radiotherapy, these investigations have primarily focused on geometric coverage^11^, ^12^. However, in lattice therapy, where the beamlet edges are relatively large, geometric coverage is rarely problematic. Significant tumor motion can markedly alter the dose differential and spatial separation between high‐ and low‐dose regions, potentially compromising clinical outcomes. This study therefore aimed to analyze the effects of liver motion on lattice radiotherapy (LRT), and to evaluate the importance of motion management in LRT using dosimetric indices.

## 2 MATERIALS AND METHODS

### 2.1 Model establishment

This study enrolled 24 patients with liver cancer who completed radiotherapy treatment were randomly selected. The study protocol was approved by the Ethics Committee of the Affiliated Cancer Hospital of Shandong First Medical University (approval number: SDTHEC202505053.

All patients underwent 4D‐computed tomography (CT) for localization, with the scan covering the entire liver volume. Respiratory motion amplitude varies across liver segments,^13^ and is greatest in the superior‐inferior (SI) direction within the liver. Our study objective was to analyze the impact of respiratory motion on lattice dose distribution; therefore, segments VII and VIII, exhibiting the largest motion amplitude on free‐breathing scans, were designated as the regions of interest (ROI). According to the Couinaud liver segmentation system,^14^ segments VII and VIII are located in the right posterior superior region and the right anterior superior regions of the liver, respectively. These segments are situated in the superior portion of the right lobe and exhibit the greatest amplitude of respiratory motion owing to their proximity to the diaphragm.^13^ Given our core objective of quantifying the maximum potential detrimental impact of respiratory motion on LRT dose distribution, selecting segments VII and VIII (with the largest motion amplitude) was considered to most prominently reveal motion‐induced dose degradation, which helps to establish a ‘worst‐case scenario’ reference for clinical motion management.

**FIGURE 1:**
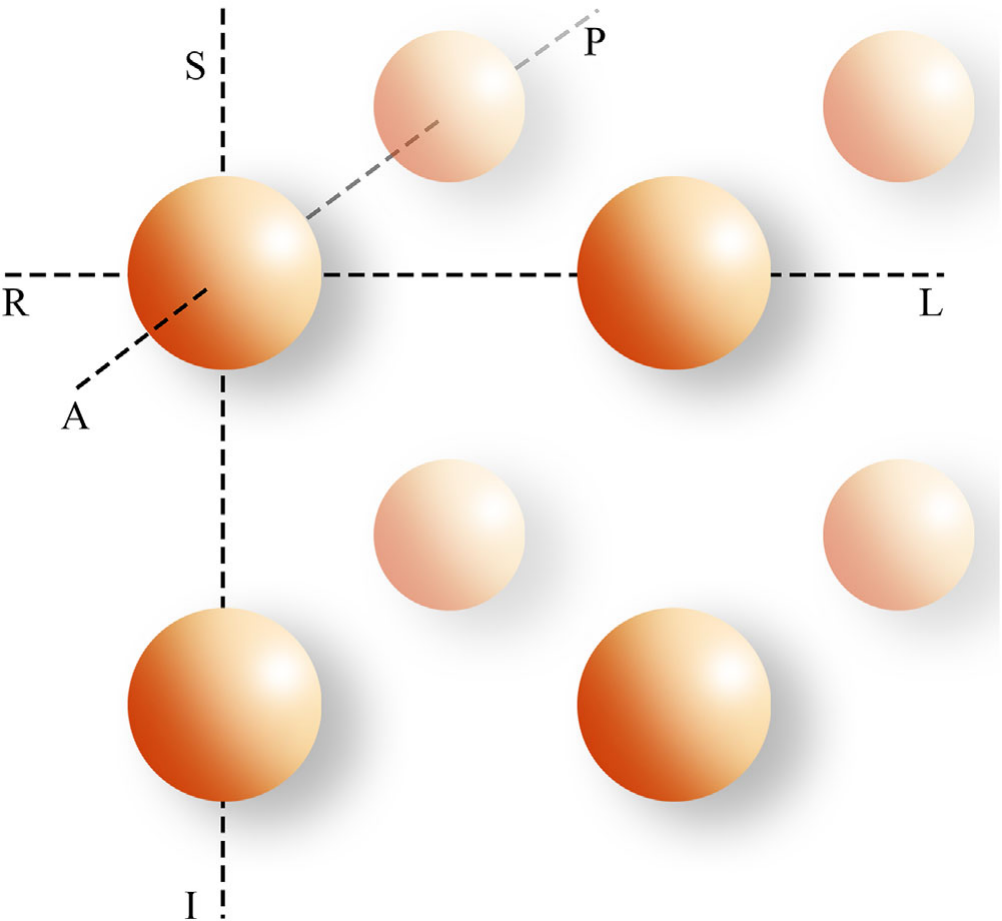
Vertices array model.

A 2×2×2 vertex array model (Figure 1) was constructed at the center of the ROI. In this model, the vertices were uniformly distributed across a defined area. Based on previous research^3^, ^15^ and clinical experience in our institution, a moderate parameter configuration was chosen, with a vertex diameter of 1 cm and an intervertex spacing of 3 cm. While varying the number, diameter, or spacing of vertices would certainly affect the absolute values of dosimetric parameters, such as valley‐to‐peak dose ratio (VPDR), the fundamental trends and conclusions regarding respiratory motion effects were expected to remain consistent, even though the magnitude of motion‐induced degradation may vary with different parameter configurations.

### 2.2 Plan design

All 4D‐CT scans were conducted using a Philips Brilliance Big Bore CT simulator. Respiratory motion was monitored using an abdominal pressure belt equipped with infrared markers. The scan covered the entire liver volume, with a slice thickness of 3 mm. The respiratory cycle was divided into 10 phases (0%–90%), and images were reconstructed using a phase‐binning algorithm. The free‐breathing CT comprised a conventional 3D CT scan acquired during the same simulation session, for which the use of identical reconstruction algorithms and slice thicknesses minimized potential dose calculation biases introduced by differences in CT protocols.

A Vital Beam accelerator system (Varian Medical Systems, Palo Alto, CA, USA) was selected as the target device. This selection was primarily based on the Vital Beam's capability for the 6 MV flattening‐filter‐free (FFF) energy mode and its collimator auto‐following function, which enables a steeper dose fall‐off and better reduction of the low‐dose bath volume. Using free‐breathing CT data (conventional 3D free‐breathing CT acquired without respiratory phase sorting, with a slice thickness of 3 mm reconstructed using a standard algorithm), volumetric modulated arc therapy (VMAT) was administered. The isocenter was positioned at the center of the vertex model. The plans were optimized using the Photon Optimizer (PO, version 16.1), and calculated using the Anisotropic Analytical Algorithm (AAA, version 16.1), with a calculation grid size of 0.1 cm. The collimator angles were set to 20°, 70°, 85°, and 345°. Auxiliary ring structures and additional avoidance sectors were defined to achieve the desired dose distribution required for the lattice structure. The prescribed dose was set at 50 Gy, delivered in two fractions. The planning objective specified that 50% of the total combined volume of all vertices should receive this prescribed dose. No maximum dose constraint was applied to the vertices to maximize the peak dose.

For the same treatment fraction, the dose from the target plan was divided into 10 equal subcomponents. The planning CT scan was rigidly registered with each phase image of the 4D‐CT. Based on the displacement measured at the time point corresponding to each 1/10th dose subcomponent within the 4D‐CT registration, an isocenter shift was applied to the dose distribution calculated for the respective 4D‐CT phase. Finally, the dose distributions from all of the phase‐specific plans were summed to obtain the delivered dose distribution, which represented the intra‐fractional dose variation. To facilitate a more accurate comparison of the low‐dose bath volumes, the summed delivered dose distribution was normalized to match the prescribed dose coverage value.

### 2.3 Valley‐to‐Peak Dose Ratio (VPDR)

The VPDR is a crucial metric for evaluating the dose distribution characteristics of the lattice geometries. Although numerous calculation formulae for VPDR have been established in various prior studies,^16^ a specific formula was selected for this simulation‐based investigation.  Specifically, the dose profile curves traversing the spherical centers of adjacent vertices (as illustrated) were analyzed. From these profiles, the D_peak_ within the high‐dose vertex regions and D_valley_ within the lower‐dose regions between the vertices were determined. The VPDR was subsequently calculated using the following formula:^17^

$$VPDR=\frac{D_{valley}}{D_{peak}}$$

Twelve dose profiles were acquired across the antero‐posterior (AP), left‐right (LR), and SI directions. The mean VPDR was calculated for each direction.

### 2.4 Planning metrics

Dosimetric metrics were extracted from the treatment planning system to comprehensively evaluate the plan performance. Specifically, the maximum (D_max_) and mean (D_mean_) doses to the eight vertices were analyzed, along with the dose distribution surrounding these vertices. To assess normal tissue sparing, the absolute volumes that received at least 40%, 20%, and 10% of the prescribed dose (V_40%_, V_20%_ and V_10%_, respectively) were extracted. The impact of respiratory motion on adjacent organs at risk (OARs), including the stomach, liver, and spinal cord, was also evaluated by comparing the dose‐volume metrics between the target and delivered plans. Furthermore, the differences among the target, delivered, and normalized delivery plans were evaluated.

## 3 RESULTS

### 3.1 Intra‐fraction motion error

Quantitative data describing the liver motion during a single respiratory cycle are presented in Table 1.  Liver motion errors result from combined displacements in three‐dimensional directions (LR, AP, and SI). The magnitude of motion was calculated using the following formula:

$$\begin{array}{ccc} & & motion\;error=\\ & & \sqrt{{\left(\left|max_{▵LR}-min_{▵LR}\right|\right)}^{2}+{\left(\left|max_{▵AP}-min_{▵AP}\right|\right)}^{2}+{\left(\left|max_{▵SI}-min_{▵SI}\right|\right)}^{2}} \end{array}$$

**TABLE 1:** Motion amplitude values of the 24 cases.

Case number	Motion amplitude (mm)			Motion error (mm)
	LR	AP	SI	
1	1.63	3.82	3.36	5.34
2	2.15	2.89	4.48	5.75
3	2.03	3.72	3.92	5.77
4	2.63	3.24	4.27	5.97
5	1.84	3.36	4.62	6.00
6	2.15	2.86	5.16	6.28
7	2.05	5.39	2.94	6.47
8	2.06	2.37	5.72	6.53
9	1.70	4.91	4.33	6.76
10	1.66	3.78	5.45	6.84
11	2.05	3.47	5.67	6.95
12	1.95	3.47	5.91	7.12
13	2.51	4.30	5.17	7.18
14	1.28	4.11	6.16	7.52
15	2.18	5.36	5.81	8.20
16	2.93	5.39	6.35	8.83
17	1.86	6.46	5.84	8.90
18	1.91	4.81	8.23	9.72
19	1.88	3.62	9.34	10.19
20	1.54	5.47	9.31	10.91
21	2.02	5.78	10.72	12.34
22	1.87	4.30	13.73	14.51
23	2.75	7.70	13.09	15.43
24	2.37	9.88	13.15	16.62
Mean	2.04	4.60	6.78	8.59

The motion magnitude across all patients (mean ± standard deviation) was 2.04±0.39 mm in LR, 4.60±1.68 mm in AP, and 6.78±3.15 mm in SI directions. On average, the displacement in the SI direction was significantly larger than those in both the LR and AP directions.

### 3.2 Vertices dose analysis

**FIGURE 2:**
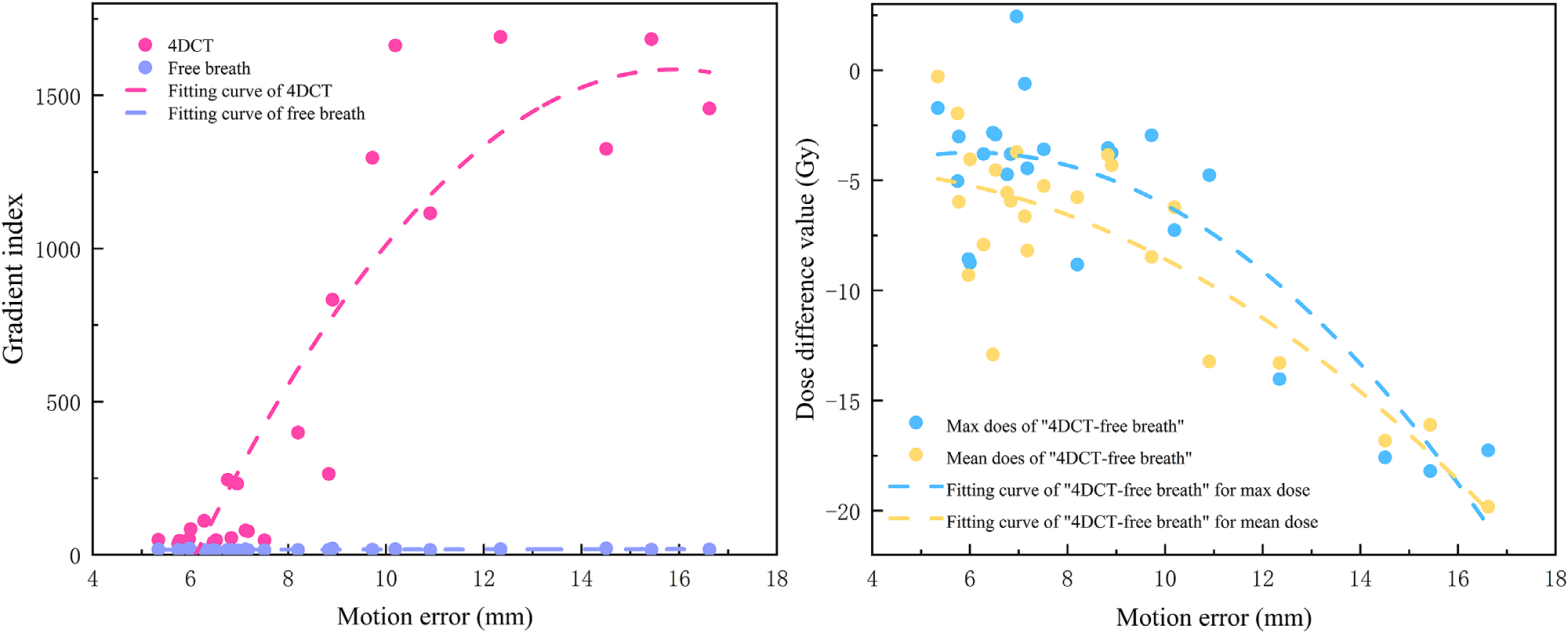
Vertices dose differences with motion error, including the GI for vertices (left) and the difference between the max and mean doses of the vertices (right).

Analysis using 4D‐CT for LRT configurations revealed that respiratory motion significantly affected both the dose distribution and homogeneity at the vertices.  The D_mean_ delivered to the vertices decreased from 50.19±0.27 Gy in the target plan to 42.26±4.98 Gy in the delivered plan.  Similarly, the D_max_ decreased from 58.94±0.79 Gy (target plan) to 52.63±5.48 Gy (delivered plan).  As illustrated in Figure 2 and Table 2, the D_mean_ and D_max_ delivered to the vertices exhibited an exponential decrease with increasing motion errors, while a rapid decrease in both D_mean_ and D_max_ was observed when the motion error exceeded 8 mm.

**TABLE 2:** Vertices dose of free breath and 4D‐CT.

Parameter	Free breath	4D‐CT	Difference
D_max_ (Gy)	58.94 ± 0.79	52.63 ± 5.48	−6.22 ± 5.46
D_mean_ (Gy)	50.19 ± 0.27	42.26 ± 4.98	−7.92 ± 4.97

### 3.3 VPDR

**FIGURE 3:**
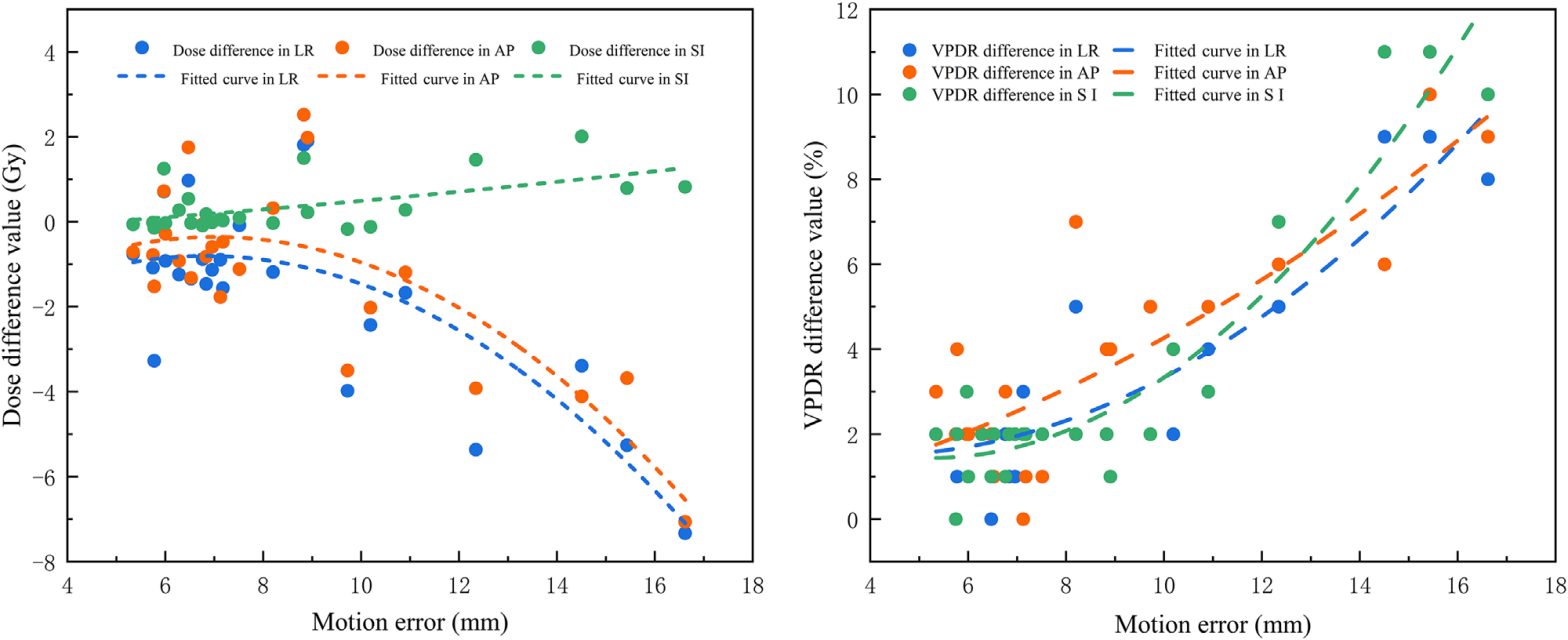
D_valley_ and VPDR differences with motion error, including the D_valley_ values for vertices (left) and VPDR difference for vertices (right).

Owing to the interdependence of dose distributions resulting from three‐dimensional motion, assessing the impact solely along a single motion direction lacks clinical relevance. Consequently, the VPDR measurements were performed in the AP, LR, and SI directions. The results are summarized in Figure 3 and Table 3.

**TABLE 3:** D_valley_ and VPDR for free breath and 4D‐CT in LR, AP, and SI.

Parameters	Free breath			4D‐CT		
	LR	AP	SI	LR	AP	SI
D_valley_ (Gy)	23.92 ± 1.16	23.77 ± 1.20	8.42 ± 2.50	22.26 ± 2.59	22.58 ± 2.56	8.80 ± 2.65
VPDR	0.41 ± 0.02	0.41 ± 0.01	0.14 ± 0.04	0.44 ± 0.03	0.45 ± 0.02	0.18 ± 0.06

In the LR and AP directions, D_valley_ exhibited comparable trends and magnitudes.  No significant variation in D_valley_ was observed in any direction when the motion error remained below 8 mm.  With an increasing motion error, D_valley_ in the LR and AP directions decreased exponentially, whereas a marginal increase occurred in the SI direction. At 16 mm displacement, the D_valley_ in LR and AP directions decreased by 7.06 Gy and 7.33 Gy respectively (both representing reductions exceeding 14%), while the SI direction showed a maximum increase of 2.01 Gy (4% increment).

**FIGURE 4:**
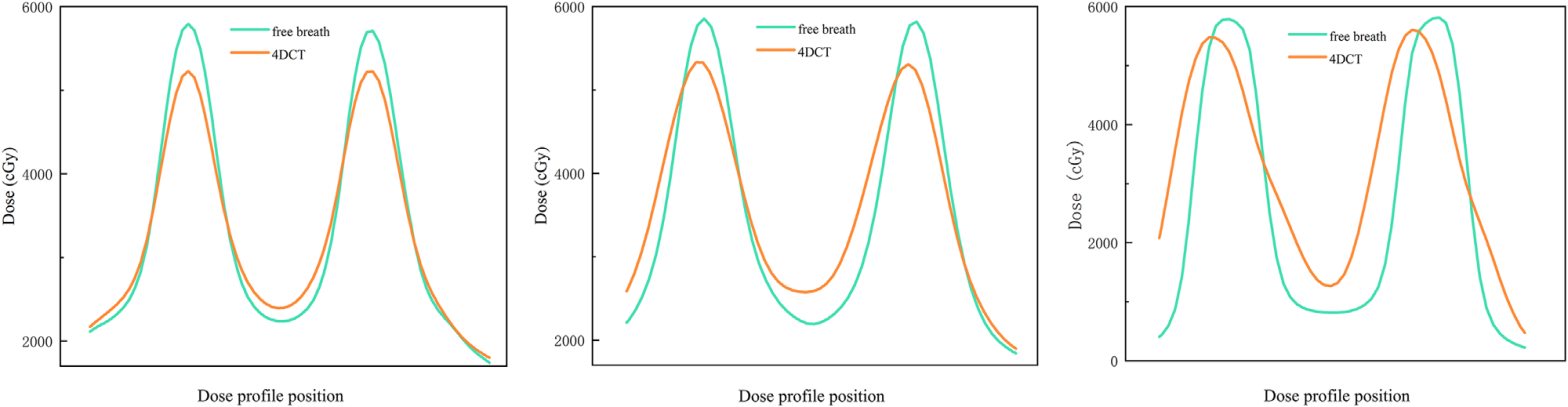
Dose profile in the LR (left), AP (middle), and SI (right) directions.

VPDR alterations demonstrated a proportional relationship with motion errors in all three directions.  Consistently, lower VPDR values were observed in the SI direction than in the LR and AP directions. When the motion error exceeded 8 mm, the VPDR in the SI direction increased at a significantly higher rate than that in the LR/AP directions, exhibiting exponential growth. Within a motion error of 5 mm to 16 mm, the average VPDR in LR and AP directions increased from 0.41±0.02 and 0.41±0.01 of the target plan to 0.44±0.03 and 0.45±0.02 of the delivered plan, respectively (Figure 4). Compared with the VPDR of the target plan, the maximum increases in the VPDR were 9% and 10% of the delivered plan, respectively. The average VPDR in the SI direction increased from 0.14±0.04 (target plan) to 0.18±0.06 (delivered plan), with a maximum increase of 11%.

### 3.4 Low dose distribution analysis

**FIGURE 5:**
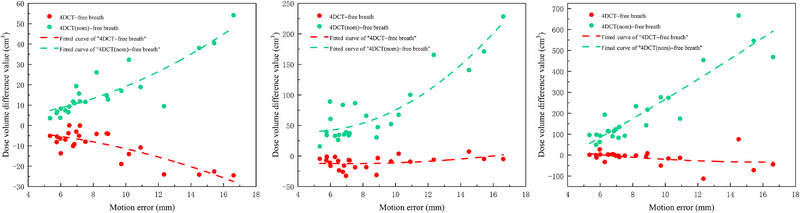
V_40%_, V_20%_, and V_10%_ differences with motion errors: V_40%_ (left), V_20%_ (middle), and V_10%_ (right).

Analysis of the low‐dose regions (Figure 5, Table 4) demonstrated that respiratory motion induced considerable reductions in V_40%_, V_20%_, and V_10%_ within the delivered plan compared to the target plan.

**TABLE 4:** Dose volume for the low dose regions.

Parameter	Free breath	4D‐CT	4D‐CT (nom)
V_40%_ (cm^3^)	48.56 ± 3.80	38.73 ± 8.28	65.10 ± 13.79
V_20%_ (cm^3^)	203.27 ± 21.27	192.64 ± 21.86	270.73 ± 64.41
V_10%_ (cm^3^)	719.92 ± 126.54	709.66 ± 124.27	924.51 ± 236.73

**TABLE 5:** Dose volume for OARs.

Parameter		Free breath	4D‐CT	4D‐CT (nom)	*P*_1_	*P*_2_
OARs	Index (Gy)					
Liver	D_mean_	4.44 ± 0.7	4.54 ± 1.01	5.17 ± 1.03	0.789	0.019
Spinal Cord	D_max_	8.07 ± 1.61	7.31 ± 1.65	8.55 ± 1.67	0.117	0.303

^#^ *P*_1_ = free breath vs. 4D‐CT; *P*_2_ = free breath vs. 4D‐CT (no).

Regardless of the magnitude of motion error, the delivered plan always had a smaller volume of low‐dose regions than the target plan. Within a motion error range of 5 mm to 16 mm, the average V_40%_, V_20%_, and V_10%_ of the delivered plan were each approximately 10 cm^3^ less than those of the target plan, indicating that the lower the dose region, the less it is affected by motion. To more clearly analyze the impact of motion error on the low‐dose regions, we normalized the delivered plan such that it had the same prescription coverage volume as the target plan. We found that motion error had a significant impact, with V_40%_, V_20%_ and V_10%_ potentially increasing by a minimum of 3.59 cm^3^, 15.85 cm^3^ and 92.29 cm^3^, respectively.

### 3.5 OARs analysis

Respiratory motion also affects the dose distribution to adjacent OARs (Table 5). Analysis of doses to the OARs revealed that motion‐induced dose variations in the OARs were patient‐specific and dependent on the relative position between the liver target and each OAR. Overall, the maximum actual delivered dose to the spinal cord decreased by 9.4%, whereas the normalized dose distribution increased in volume, overlapping with the OARs, with the average liver dose increasing by 0.73 Gy. The kidneys exhibited minimal dose changes (<0.2%) because of their relatively fixed positions and distances from the vertex target.

Owing to the superior location of the target volume within liver segments VII and VIII, these organs were consistently distant from high‐dose regions. Dosimetric analysis confirmed that the received dose was negligible and exhibited minimal variation between the plans, rendering it clinically and statistically insignificant.

### 3.6 Influence of intervertex spacing on motion sensitivity

**FIGURE 6:**
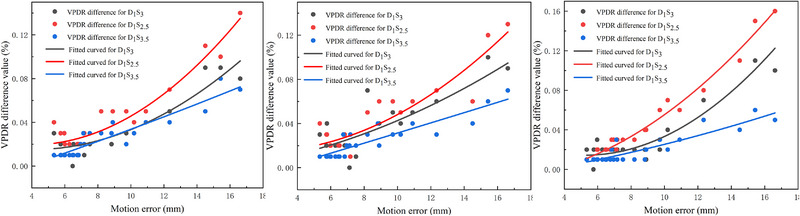
VPDR differences with motion error for various spacings, including LR (left); AP (middle), and SI (right). # D_1_S_3_: Vertex diameters (1.0 cm with a spacing of 3.0 cm; D_1_S_2.5_: Vertex diameters (1.0 cm with a spacing of 2.5 cm; D_1_S_3.5_: Vertex diameters (1.0 cm with a spacing of 3.5 cm;

To investigate the impact of LRT geometry on motion sensitivity, we conducted additional analyses comparing intervertex spacings of 2.5 cm and 3.5 cm to our baseline 3.0 cm model. As expected, the baseline VPDR in the static plan decreased with larger spacing owing to the reduced penumbral overlap. Crucially, the magnitude of motion‐induced VPDR degradation was space‐dependent. As presented in Figure 6, the increase in VPDR due to respiratory motion was most pronounced for the 2.5 cm spacing, and least pronounced for the 3.5 cm spacing. In contrast, the changes in the low‐dose bath volumes (V_40%_, V_20%_, and V_10%_) and OARs were consistent across different spacings, as these regions were characterized by shallow gradients, regardless of the configuration.

## 4 DISCUSSION

The bystander effect^18^ may play a role in lattice therapy, making intercellular communication between the high‐ and low‐dose regions crucial. Motion can alter the locations of these dose regions, potentially disrupting the communication pathways between cells, thereby attenuating the bystander effect. Previous studies have investigated the impact of motion on SFRT plan quality. For example, Naqvi et al. ^19^ simulated different tumor motion patterns using a physical block, and investigated their effects on GRID therapy. Similarly, Yang et al. ^20^ evaluated the influence of respiratory motion on the delivered dose in proton pencil beam scanning (PBS) for the delivery of FLASH radiotherapy through simulations and phantom measurements.

**FIGURE 7:**
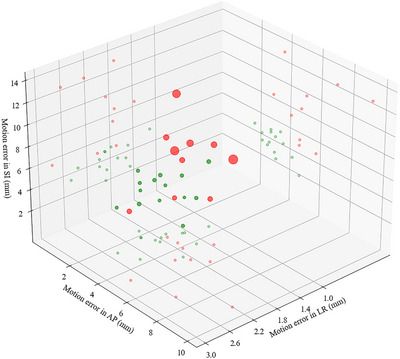
Scatter plot of the motion errors in three directions.

The findings of this study highlight the significant adverse impact of respiratory motion on the dose distribution in LRT for liver tumors. Analysis of liver motion during a single respiratory cycle revealed substantial inter‐patient variability in motion amplitude, with the greatest displacement observed along the SI direction. This aligns with the results of prior studies demonstrating predominantly SI‐directed liver motion during respiration.^13^ The calculated combined 3D displacement (representing the motion error) averaged 8.59 mm across all patients, underscoring the necessity for motion management in clinical practice. Figure 7 presents a scatter plot of the respiratory‐induced errors for 24 patients, for which red bubbles denote cases with motion errors exceeding 8 mm, and green bubbles indicate cases below this threshold. Both Figure 6 and Table 1 demonstrate that the LR directional errors never exceeded 3 mm, contributing negligibly to the total motion error; the 3D motion errors exceeded 8 mm when either the AP directional errors exceeded 5 mm or the SI directional errors exceeded 6 mm.

Respiratory motion induces a significant degradation in dose distributions at the vertices, manifesting as substantial reductions in both the mean and maximum delivered vertex doses compared with the planned distribution. When the motion errors exceeded 8 mm, the dose exhibited an exponential decay, indicating that even modest motion errors may cause critical target underdosage and potentially compromise the efficacy of LRT. The exponential dose‐motion relationship underscores the importance of rigorous respiratory motion mitigation to ensure adequate tumor coverage. Our analysis further revealed that motion reduced the low‐dose bath volumes (V_40%_, V_20%_, and V_10%_) in the delivered plans, demonstrating that its impact extends beyond high‐dose regions to areas essential for systemic immune stimulation^21^ and normal tissue protection via bystander effects. Conversely, when normalizing the delivered plans to match the prescription coverage volume of the planned distributions, motion errors paradoxically increased the V_40%_, V_20%_, and V_10%_ volumes, highlighting the critical need for motion management to preserve the therapeutic low‐dose bath configuration, which is vital for minimizing the RILD^22^ risk while maximizing normal tissue sparing.

The VPDR is a critical metric for characterizing dose distributions in grid geometries,^23^ for which spatial fractionation heterogeneity is quantified by the VPDR. This ratio is governed by vertices diameter (0.5–1.5 cm at isocenter), center‐to‐center spacing (2–5 cm), grid arrangement, and photon energy.^15^ VPDR depends on primary penumbral overlap^24^: when the minimum edge‐to‐edge spacing (smin) is < 2Re (lateral electron range), secondary electrons cause significant penumbral overlap. Under these conditions, even though secondary electrons from higher‐energy photons are predominantly forward‐directed, their larger Re results in a lower VPDR at or slightly beyond the depth of maximum dose (D_max_). Conversely, at smin ≥ 2Re, VPDR is dominated by scattering‐tail superposition. In this scenario, the VPDR is primarily governed by the superposition of the scatter tails from adjacent beams. For all energies, the VPDR decreases with depth as the valley doses increase from cumulative scatter, particularly at lower energies.^25^ Although modifying parameters such as the vertex number, diameter, or spacing may affect the absolute values of the VPDR and other dosimetric parameters, the fundamental trend of respiratory motion‐induced degradation of spatial fractionation is expected to persist across different LRT configurations. Crucially, this study reveals that respiratory motion increases VPDR (especially SI), homogenizing distributions and eroding the “peak‐valley” effect underlying the biological advantages of SFRT. Exponential VPDR deterioration beyond 8 mm of SI motion underscores its disproportionate impact compared with AP/LR directions, necessitating motion management strategies specifically optimized for SI motion to preserve therapeutic dose heterogeneity.

The impact on adjacent OARs, although generally less pronounced than that on the target volume, warrants consideration. The observed dose variations in the liver and spinal cord, although modest on average, could be clinically significant for individual patients depending on their specific anatomy and baseline OAR doses. This finding reinforces the importance of patient‐specific motion management approaches.

The findings of this study could have significant clinical implications for the implementation of LRT for liver tumors. Substantial respiratory motion‐induced dose degradation necessitates the incorporation of motion management techniques, such as breath‐holding,^26^ abdominal compression,^27^ and real‐time tumor tracking,^28^ into clinical workflows to minimize the intrafraction motion and ensure dosimetric fidelity. Complementarily, 4D‐CT imaging and motion‐adaptive planning^29^ can further enhance precision by explicitly accounting for respiratory motion during treatment planning. Critically, the directional anisotropy of motion errors, compounded by the steep source‐to‐surface distance variations and differential beam penetration^30^ inherent to SFRT geometries, requires tighter dose‐fall‐off constraints along the SI axis. To mitigate these effects, treatment optimization should strategically expand the aperture placements in the LR/AP directions, capitalizing on their greater inherent motion tolerance, while maintaining therapeutic heterogeneity through motion suppression in the SI dimension.

Our findings indicate that motion management is particularly crucial for tumors located in segments VII and VIII, which experience the greatest respiratory motion. The pronounced dose degradation observed in these segments indicates that LRT treatments in these regions may be more vulnerable to motion‐induced errors than those in other liver segments with smaller motion amplitudes.

## 5 LIMITATIONS AND FUTURE DIRECTIONS

This study has several limitations that should be considered when interpreting the results. First, although this study quantitatively demonstrated the significant physical degradation of LRT dose distribution caused by respiratory motion, it did not establish any corresponding radiobiological consequences. Second, the respiratory motion model used in this study, based on 4D‐CT, inherently assumes regular and periodic breathing patterns, which may underestimate the dose degradation encountered in patients with irregular or erratic breathing. Third, this study was designed to identify and quantify motion issues during the LRT. The next research step will be to rigorously evaluate and compare the effectiveness of various motion management strategies (e.g., respiratory gating, abdominal compression, and real‐time tracking) at mitigating these effects. Finally, as a computational dose simulation study, this study did not model the cumulative impact of other treatment delivery uncertainties. Validating these complex motion‐affected dose distributions by conducting measurements in anthropomorphic phantoms is a necessary next step for translating these findings into clinical applications.

## 6 CONCLUSION

This study demonstrated that respiratory motion significantly degrades the spatial dose heterogeneity, a fundamental characteristic of LRT, in the treatment of liver tumors. Collectively, these findings underscore the necessity for robust motion management to ensure dosimetric accuracy, preserve therapeutic peak‐to‐valley dose distributions, and maintain LRT ’s inherent radiobiological advantages of LRT.

## AUTHOR CONTRIBUTIONS

Kuo Li and Yong Yin contributed to the data collection and analysis and drafted the initial manuscript. Kuo Li conducted the statistical analyses and created the figures. Yong Yin and Tianyuan Dai worked together to complete the manuscript revision and design the experimental protocol. Jian Zhu and Zhenjiang Li reviewed the study design, designed research methodology, and collected funding. Tonghai Liu was involved in data analysis and provided technical support. All other authors contributed to the data collection. All the authors reviewed the results and approved the final version of the manuscript.

## CONFLICT OF INTEREST STATEMENT

The authors declare that they have no known competing financial interests or personal relationships that may have influenced the work reported in this study.

## ETHICS STATEMENT

The study protocol was reviewed and approved by the Ethics Committee of the Affiliated Cancer Hospital of Shandong First Medical University (approval number: **SDTHEC202505053**), and was confirmed to comply with the principles of the Declaration of Helsinki. As this study was retrospective in nature and involved only the analysis of fully anonymized data extracted from the treatment planning system, without any additional interventions or contact with patients, the ethics committee granted a waiver of the requirement to obtain individual patient informed consent. Only anonymized datasets were used for analysis during the study to ensure that patient privacy and data confidentiality were maximally protected.
